# Increased expression level of ANGPTL8 in white adipose tissue under acute and chronic cold treatment

**DOI:** 10.1186/s12944-021-01547-0

**Published:** 2021-09-26

**Authors:** Hossein Arefanian, Irina Al-Khairi, Nermeen Abu Khalaf, Preethi Cherian, Sina Kavalakatt, Dhanya Madhu, Aditi Mathur, Mohamed G. Qaddoumi, Fahd Al-Mulla, Jehad Abubaker, Mohamed Abu-Farha

**Affiliations:** 1 Immunology & Microbiology Department, Dasman Diabetes Institute, Dasman, Kuwait; 2 Biochemistry and Molecular Biology Department, Dasman Diabetes Institute, Dasman, Kuwait; 3 Animal and Imaging Core Facilities, Dasman Diabetes Institute, Dasman, Kuwait; 4 Special Service Facility, Dasman Diabetes Institute, Dasman, Kuwait; 5 Pharmacology and Therapeutics Department, Faculty of Pharmacy, Kuwait University, Kuwait City, Kuwait; 6 Genetics and Bioinformatics Department, Dasman Diabetes Institute, Dasman, Kuwait; 7 Biochemistry & Molecular Biology Unit, P.O. Box 1180, 15462 Dasman, Kuwait

**Keywords:** Angiopoietin-like proteins, ANGPTL, Adipose Tissue, Browning, Cold Treatment, UCP1

## Abstract

**Background:**

Angiopoietin-like proteins (ANGPTL), primarily 3, 4, and 8, play a major role in maintaining energy homeostasis by regulating triglyceride metabolism. This study evaluated the level of ANGPTL3, 4, and 8 in the liver, brown adipose tissue (BAT), and subcutaneous white adipose tissue (SAT) of mice maintained under acute and chronic cold conditions.

**Methods:**

C57BL/6J mice were exposed to cold temperature (4 °C) for 10 days with food provided *ad libitum*. Animal tissues were harvested at Day 0 (Control group, *n* = 5) and Days 1, 3, 5, and 10 (cold treatment groups, *n* = 10 per group). The expression levels of various genes were measured in the liver, SAT, and BAT. ANGPTL3, 4, and 8 expressions were measured in the liver. ANGPTL4, 8, and genes involved in browning and lipid metabolism [uncoupling protein 1 (*UCP1*), lipoprotein lipase (*LPL*), and adipose triglyceride lipase (*ATGL*)] were measured in SAT and BAT. Western blotting (WB) analysis and immunohistochemistry (IHC) were performed to confirm ANGPTL8 expression in these tissues.

**Results:**

The expressions of ANGPTL3 and 8 mRNA were significantly reduced in mouse liver tissues after cold treatment (*P* < 0.05); however, the expression of ANGPTL4 was not significantly altered. In BAT, ANGPTL8 expression was unchanged after cold treatment, whereas ANGPTL4 expression was significantly reduced (*P* < 0.05). ANGPTL4 levels were also significantly reduced in SAT, whereas *ANGPTL8* gene expression exhibited over a 5-fold increase. Similarly, *UCP1* gene expression was also significantly increased in SAT. The mRNA levels of LPL and ATGL showed an initial increase followed by a gradual decrease with an increase in the days of cold exposure. ANGPTL8 protein overexpression was further confirmed by WB and IHC.

**Conclusions:**

This study shows that exposure to acute and chronic cold treatment results in the differential expression of ANGPTL proteins in the liver and adipose tissues (SAT and BAT). The results show a significant reduction in ANGPTL4 in BAT, which is linked to improved thermogenesis in response to acute cold exposure. ANGPTL8 was activated under acute and chronic cold conditions in SAT, suggesting that it is involved in regulating lipolysis and enhancing SAT browning.

**Supplementary Information:**

The online version contains supplementary material available at 10.1186/s12944-021-01547-0.

## Background

Recently, cold exposure has emerged as a potential therapeutic approach to target metabolic dysregulation [1]. It is known that exposure to cold temperatures leads to an increase in energy expenditure via the activation of thermogenic fat metabolism and glucose utilization. This, in turn, improves insulin sensitivity and energy homeostasis [2]. The angiopoietin-like protein (ANGPTL) family members, specifically ANGPTL 3, 4, and 8, are involved in regulating lipid metabolism [3]. ANGPTL3 is exclusively expressed in the liver, whereas ANGPTL4 and 8 are expressed in the liver, adipocytes, and other tissues [4–7]. A well accepted ANGPTL3-4-8 model for the role of ANGPTL proteins hypothesizes that these three proteins regulate lipoprotein lipase (LPL) activity in a tissue-specific manner depending on the nutritional status [3, 6]. During the fasting/fed state, the level of ANGPTL3 is stable. However, ANGPTL4 and 8 respond differentially to food—ANGPTL4 is induced by fasting, whereas the level of ANGPTL8 is increased in the fed state [6]. During fasting, the induction of ANGPTL4 leads to the inhibition of LPL activity in adipose tissue and directs triglycerides (TGs) to cardiac and skeletal muscles and away from fat storage. In contrast, feeding induces the expression of ANGPTL8, which forms a complex with ANGPTL3 to inhibit LPL activity in cardiac and skeletal muscles, directing TGs for storage in adipocytes [6]. Furthermore, it has been shown that during the fed state, ANGPTL8 forms a complex with ANGPTL4 in adipocytes. This complex ensures the induction of LPL activity, which results in increased fatty acid (FA) uptake by adipocytes [7]. In addition to its role in regulating lipid metabolism in the circulation, ANGPTL8 may contribute to adipocyte differentiation [8].

Adipose tissue is not only a fat storage depot but also an important endocrine organ that secretes various adipokines involved in regulating metabolism [9–11]. There are two primary types of adipose tissue, white adipose tissue (WAT), classified as subcutaneous (SAT) and visceral (VAT) based on their distribution in the body, and brown adipose tissue (BAT) [12]. WAT primarily functions as a lipid storage tissue and is the primary source of energy, particularly during fasting [13]. In contrast, BAT plays a major role in regulating energy expenditure during cold exposure by enhancing mitochondrial heat production via a nonshivering mechanism [9, 14]. Bartelt et al. reported that short-term cold exposure results in increased BAT activity, which enhances TG uptake in a process that relies on LPL enzyme activity [15].As the ANGPTL protein family members are important regulators of TG metabolism in the adipose tissue, several studies have focused on the role of ANGPTL4 in cold treatment [16, 17]. However, studies on the effect of cold on ANGPTL8 in these tissues are lacking. The primary objective of the present study was to evaluate the tissue-specific expressions of ANGPTL8, 3, and 4 in the liver, SAT, and BAT under acute and chronic cold conditions.

## Methods

### Animal studies and cold exposure

C57BL/6J mice were purchased from the Jackson laboratory (Bar Harbor, ME, USA), bred, and kept in a barrier animal facility at the Dasman Diabetes Institute (DDI) under constant temperature (22 °C ± 1 °C) and humidity in a 12-h controlled dark/light cycle (lights on: 7:00 am to 7:00 pm). All mice were provided with *ad libitum* access to drinking water and a normal chow diet containing 6.2 % calories as total fat (EURodent Diet 14 %, 5LF2, LabDiet, St. Louis, MO, USA).

In total, 45 male mice (age 12 weeks old) were used for this study. The mice were randomly allocated into two groups—cold exposure group (*n* = 40) and room temperature control group (*n* = 5). For cold exposure, 40 male mice were singly housed in pre-chilled cages with minimal bedding, a 12-hour dark/light cycle, and *ad libitum* access to water and chow diet in a cold controlled environment at 4 °C. The mice (*n* = 10 in each group) were sacrificed at the end of Days 1, 3, 5, and 10 post-cold induction (labeled Day 1, Day 3, Day 5, and Day 10, respectively). The control group included mice that were singly housed, but kept at room temperature with no cold exposure, minimum bedding, a 12-hour dark/light cycle, and *ad libitum* access to water and chow diet (labeled as Day 0).

Weight and food intake were measured for each animal prior to euthanasia. The mice were sacrificed under nonfasting conditions. Liver biopsies and fat deposits, including SAT and BAT, were collected for further analysis. All procedures were approved by the DDI Animal Care and Ethics Committee according to international regulations and laws on animal research.

A preliminary controlled experiment was performed to rule out incidences of stress and hypothermia. For this, 10 mice were maintained under cold conditions for the abovementioned time points, their physical activity was observed, and their rectal temperature was monitored using a microprobe (50-7221 F, Harvard Apparatus, Holliston, MA, USA).

### RNA isolation and quantitative real-time polymerase chain reaction

Dissected tissues were immediately placed in RNAlater RNA Stabilization Reagent (Qiagen, Hilden, Germany) and stored at − 80 °C for subsequent RNA extraction. Total RNA was extracted and isolated from tissues using TRIzol reagent (Cat. No. 15,596,026, Invitrogen, Carlsbad, CA, USA) following the manufacturer’s instructions. For analyzing mRNA expression, the extracted samples were first quantified to assess quality and concentration using the Epoch microplate spectrophotometer (Biotek Instruments, Inc., Winooski, VT, USA). cDNA was synthesized from 1 µg RNA using a High-Capacity cDNA Reverse Transcription kit (Cat. No. 4,368,814, Applied Biosystems, Foster City, CA, USA) according to the manufacturer’s instructions. Quantitative real-time polymerase chain reaction (qRT-PCR) was performed in duplicates using QuantiTect SYBR Green PCR kits (Qiagen, Inc.) and an Applied Biosystems 7500 Real-Time PCR System (ThermoFisher Scientific). Relative gene expression (normalized to 18 S rRNA) was calculated using the comparative Ct method formula, 2-ΔΔCT, and reported as mean fold change in gene expression. The following primers were used for amplification: ANGPTL3 (forward [FW]: TGCACCTTCAGAGCCAAAAT, reverse [RV]: CATTGGTTCGAAGTGATAGGTCA), ANGPTL4 (FW: ACAGTGACTTTGGTTGTGGC, RV: CTCGAGCCCATGTTTTCTGG), ANGPTL8 (FW: CTCTCTGCCTCCTGTGGAC, RV: GCTCTGTACACGCCATTGAG), uncoupling protein 1 (UCP1) (FW: CTTTGCCTCACTCAGGATTGG, RV: ACTGCC ACACCTCCAGTCATT), LPL (FW: GGAAGAGATTTCTCAGACATCG, RV: CTACAATGACATTGGAGTCAGG), adipose triglyceride lipase (ATGL) (FW: TCCCACTTTCCAAGGAT, RV: AGCTTCCTCTGCATCCTCTTC, and 18 S (FW: CTGAGAAACGGCTACCACATC, RV: GGCCTCGAAAGAGTCCTGTAT).

### Immunohistochemistry and confocal imaging

For immunohistochemistry (IHC) analysis, formalin fixed and paraffin-embedded sections (of 8-µm thickness) from SAT at each time point were used. These sections were deparaffinized and then rehydrated, followed by antigen retrieval at pH 6 using DAKO reagents (Dako, Glostrup, Denmark). Quenching of endogenous peroxidase activity was done using 3 % hydrogen peroxide solution (1 h at room temperature). The sections were then blocked, first with 5 % fat-free milk and then with 1 % bovine serum albumin. They were then incubated with the primary antibody, anti-ANGPTL8 (MAB8548, R&D systems, USA), at 4 °C overnight followed by Alexa fluor 488-conjugated secondary antibody at room temperature for 1 h (A-11,008, Invitrogen, USA, 1:100 dilution). Nuclear staining was performed using 4′,6-diamidino-2-phenylindole at 0.05 %. A Zeiss LSM 710 confocal laser scanning microscope (Zeiss, Germany) was used to acquire the fluorescent images. The images were taken at 40× magnification for each group. Zen software (Zeiss, Germany) was used to quantitate fluorescence intensities.

### Western blotting analysis

Frozen rat tissues were cut into ~ 30–40-mg pieces. Following this, 500 µl of radioimmunoprecipitation assay buffer (50 mM Tris-HCl, pH 7.5, 150 mM sodium chloride, 1 % Triton X-100, 1 mM ethylenediaminetetraacetic acid, 0.5 % sodium deoxycholate, and 0.1 % sodium dodecyl sulfate [SDS]) was added. Following homogenization using a Tissue lyser II with stainless steel beads (Cat. No. 69,989, Qiagen), the homogenates were centrifuged at 15,000 rpm at 4 °C. The supernatants were collected, and Bradford’s protein assay was used to measure protein concentration. The protein samples (20 µg) were prepared in loading buffer containing β-mercaptoethanol and loaded onto 10 % SDS-polyacrylamide gel electrophoresis gels. Samples from each time point were loaded in duplicates representing liver, SAT, and BAT tissues along with a loading control. Proteins were transferred to polyvinylidene fluoride membranes (100 V for 75 min) and blocked for 2 h at room temperature using 5 % nonfat dried milk in Tris-buffered saline containing 0.05 % Tween (TBST). The membranes were then incubated with the primary antibody, anti-ANGPTL8 (PA5-38043, Invitrogen), overnight at 4 °C. The membranes were then washed with TBST, followed by incubation with rabbit horseradish peroxidase (HRP)-conjugated secondary antibody (1:10,000 dilution) for 2 h at room temperature. Protein bands were visualized by chemiluminescence using the super-sensitivity West Femto ECL reagent (Thermo Scientific, USA) and the gels were imaged using a Versadoc 2000 (Bio-Rad, USA) system. Quantity One Software (Bio-Rad, USA) was used to measure the band intensities. Anti-GAPDH antibody (ab2302; Millipore, Temecula, CA, USA) was used as the internal control for protein loading.

### Statistical analysis

A minimum sample size of 4–6 mice per group was estimated to be sufficient for obtaining statistical significance considering an error of 0.05, power of 0.80, a percentage change in means (PC) of 20 %, and a coefficient of variation of 10–15 % (experimental variability). Values are expressed as means of duplicate experiments ± standard error of the mean for each sample. All data were analyzed, and figures were prepared using GraphPad Prism 6 (GraphPad Software Inc., California, USA). Statistical analysis was performed using an unpaired, two-tailed Student’s t*-*test to assess differences between two groups and the nonparametric Kruskal–Wallis test for multiple comparisons. *P-*values less than 0.05 were considered statistically significant.

## Results

### Animal weight and food consumption

**Fig. 1:**
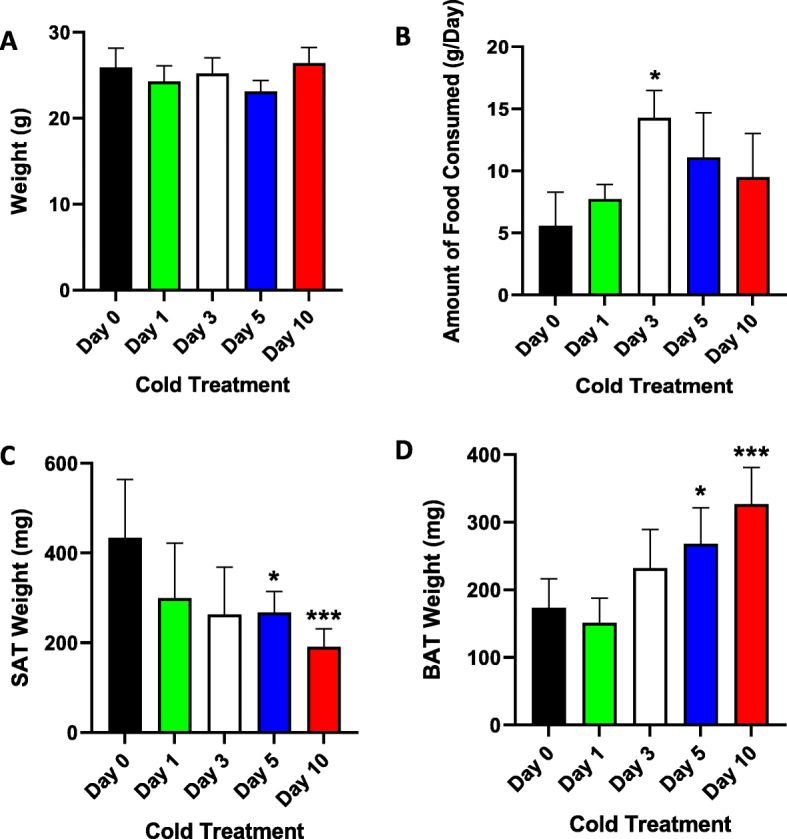
Animal weight, food consumed, and adipose tissue (SAT and BAT) weight at different time points of cold exposure (Day0 (Control-no cold exposure), Day1, Day3, Day 5, and Day10). **A**: Animal weight (g) at different days of cold treatment. **B**: Amount of food consumed daily (g) over the ten days of cold treatment. **C**: SAT weight (mg) at different days of cold treatment. **D**: BAT weight (mg) different days of cold treatment. * indicates *P* ≤ 0.05; *** indicates *P* ≤ 0.0001 as determined using Student’s t-test

Body weight and food intake were measured for each of the mice at specific time points (Day 0, Day 1, Day 3, Day 5, and Day 10). No significant change in average body weight was observed among mice at the various time points (Fig. 1 A). A significant increase in food consumption was observed following cold exposure for several days, particularly among mice at Day 3 compared with control mice (Day 0) (Fig. 1B, *P* = 0.03). Interestingly, the average SAT weight across the various time points of cold exposure was decreased compared with the SAT weight at Day 0 (Fig. 1 C, Day 5 *P* = 0.005; Day 10 *P* < 0.0001). In contrast, a significant increase in the average BAT weight was observed in mice exposed to cold over time, with the highest increase observed at Day 10 (Fig. 1D, *P* = 0.0001) compared with BAT weight at Day 0.

### Gene expression analysis in liver tissues

**Fig. 2:**
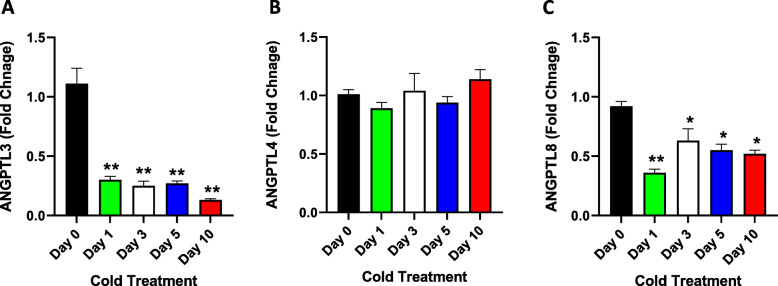
Liver ANGPTL3, ANGPTL4 and ANGPTL8 gene expression measured at Day 0 (Control no cold exposure, *n* = 5), Days 1, 3, 5 and 10 post cold treatment at 4 ^0^ C (*n* = 10 each). **A**: Expression level of ANGPTL3 in Liver as measured by RT-PCR at different time points of cold treatment. **B**: Expression level of ANGPTL4 in liver as measured by RT-PCR at different time points of cold treatment. **C**: Gene expression level of ANGPTL8 as measured by RT-PCR at different time points of cold treatment. ns indicates no significance; * indicates *P* ≤ 0.05; ** indicates *P* ≤ 0.001, as determined using Student’s t-test

Gene expression analysis of ANGPTL3, 4, and 8 was performed using the mRNA extracted from liver biopsy samples. The results showed a significant (approximately 4-fold) reduction in the expression level of ANGTPL3 at different time points (Fig. 2 A, Day 1: *P* = 0.003; Day 3: *P* = 0.001; Day 5: *P* = 0.001; and Day 10: *P* = 0.0004) compared with ANGPTL3 expression at Day 0. No significant change in the expression of ANGPTL4 was observed (Fig. 2B). A significant reduction was also observed in ANGPTL8 expression, particularly on Day 1 (Fig. 2 C, Day 1: *P* = 0.001; Day 3: *P* = 0.04; Day 5: *P* = 0.03; and Day 10: *P* = 0.02) compared to that on Day 0.

### Gene expression analysis on adipose tissue (SAT and BAT)

**Fig. 3:**
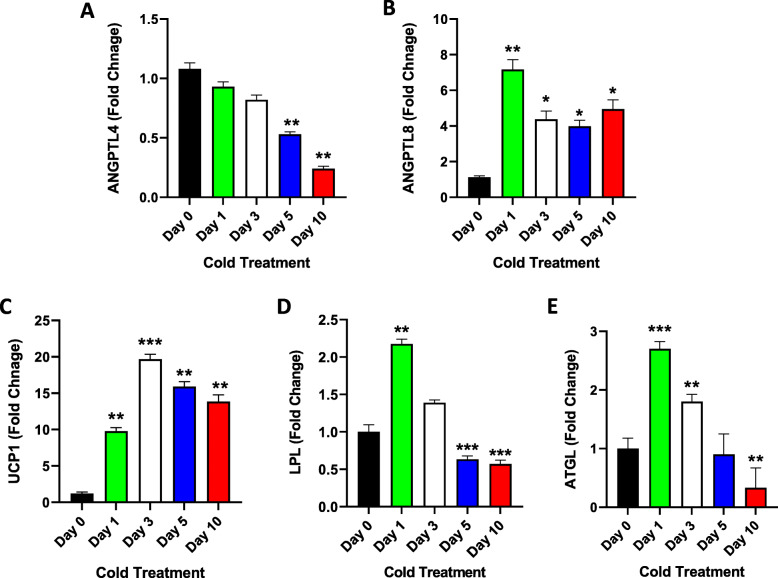
SAT UCP1, ANGPTL4 and ANGPTL8 gene expression measured at Day 0 (Control no cold exposure, *n* = 5), Days 1, 3, 5 and 10 post cold treatment at 4 ^0^ C (*n* = 10 each). **A**: Gene expression level of ANGPTL4 in SAT as measured by RT-PCR at different time points of cold treatment. **B**: Gene expression level of ANGPTL8 in SAT as measured by RT-PCR at different time points of cold treatment. **C**: Gene expression level of UCP1 in SAT as measured by RT-PCR at different time points of cold treatment. **D**: Gene expression level of LPL in SAT as measured by RT-PCR at different time points of cold treatment. **E**: Gene expression level of ATGL in SAT as measured by RT-PCR at different time points of cold treatment. * indicates P-value ≤ 0.05; ** indicates *P* ≤ 0.001; *** indicates *P* ≤ 0.0001 as determined using Student’s t-test

Gene expression analysis was also performed on mRNA extracted from SAT and BAT. Gene expression analysis of ANGPTL4 using mRNA extracted from SAT biopsies revealed a decrease in its expression level over time with cold exposure. The most significant decrease was observed on Days 5 and 10 of cold exposure with a ~ 2-fold and ~ 3-fold change, respectively (Fig. 3 A, *P* < 0.001). In contrast, a significant increase was observed in ANGPTL8 expression in SAT, with the highest increase observed on Day 1 of cold exposure (7.2-fold compared with that on Day 0) (Fig. 3B, *P* < 0.001). The expression of *UCP1*, a gene known to play an essential role in the process of adipose tissue browning, was significantly increased in SAT across the various time points of cold exposure. Particularly, a 20-fold increase in gene expression was observed on Day 3 compared to that on Day 0 (Fig. 3 C, *P* < 0.0001).

Gene expression analysis was also performed on two of the major genes that regulate lipolysis: *LPL* and *ATGL*. In SAT, LPL mRNA expression was significantly increased (2-fold) at Day 1 of cold exposure (*P* = 0.003), followed by a significant reduction over Days 3, 5, and 10 of cold exposure (Fig. 3D, Day 3: *P* = 0.056, Day 5: *P* = 0.0001, Day 10: *P* < 0.0001). Gene expression analysis of ATGL in SAT showed a similar pattern, i.e., a significant increase (~ 3-fold) on Day 1 (*P* < 0.0001), followed by a gradual decrease in mRNA expression through Days 3, 5, and 10, with the highest reduction observed on Day 10 of cold exposure compared to that on Day 0 (Fig. 3E, Day 3: *P* = 0.002, Day 5: *P* = 0.3, Day 10: *P* = 0.004).

**Fig. 4:**
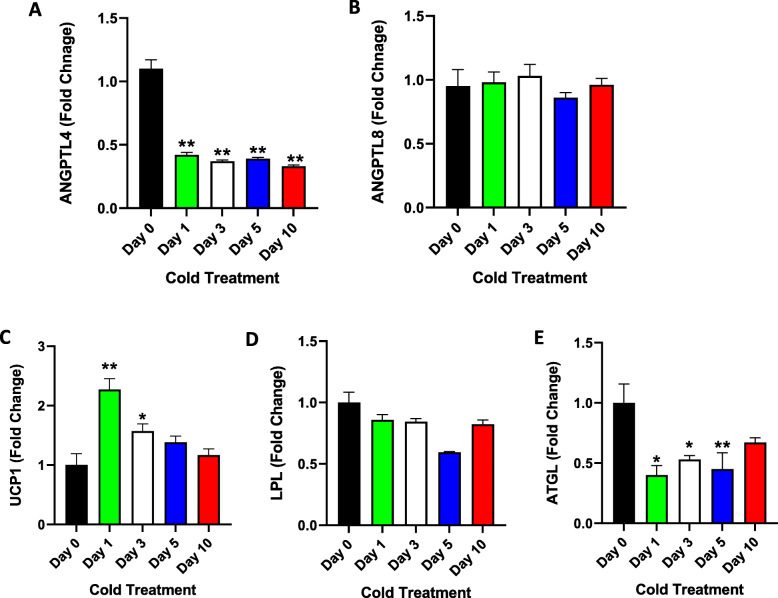
BAT UCP1, ANGPTL4 and ANGPTL8 gene expression measured at Day 0 (Control no cold exposure, *n* = 5), Days 1, 3, 5 and 10 post cold treatment at 4 ^0^ C (*n* = 10 each). **A**: Gene expression level of ANGPTL4 in BAT as measured by RT-PCR at different time points of cold treatment. **B**: Gene expression level of ANGPTL8 in BAT as measured by RT-PCR a at different time points of cold treatment. **C**: Gene expression level of UCP1 in BAT as measured by RT-PCR at different time points of cold treatment. **D**: Gene expression level of LPL in BAT as measured by RT-PCR at different time points of cold treatment. **E**: Gene expression level of ATGL in BAT as measured by RT-PCR at different time points of cold treatment. * indicates P-value ≤ 0.05; ns indicates no significance; ** indicates *P* ≤ 0.001; *** indicates *P* ≤ 0.0001 as determined using Student’s t-test

Gene expression analysis of ANGPTL4 and 8 in BAT was also performed. A significant decrease in ANGPTL4 levels was observed beginning on Day 1 of cold exposure (~ 2-fold change, Fig. 4 A, *P* < 0.001). No change in the expression of ANGPTL8 was observed in BAT at any time point of cold exposure (Fig. 4B). A significant increase in UCP1 expression was observed on Day 1 of cold exposure, with a 3.2-fold increase compared with Day 0 (*P* < 0.05). This was followed by a decrease in the expression to normal levels over time with cold treatment (Fig. 4 C). Furthermore, there was no significant change in LPL mRNA expression over the course of cold treatment (Fig. 4D**).** Interestingly, a significant reduction (~ 2-fold) in the mRNA expression of ATGL was observed on Day 1 of cold exposure, and this level remained constant throughout the entire period of cold treatment (Fig. 4E**)**.

### ANGPTL8 expression measured by WB analysis and IHC

**Fig. 5:**
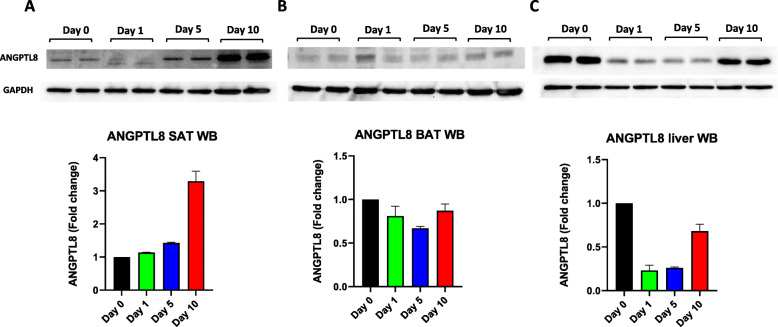
Western blotting analysis of ANGPTL8 protein level in SAT, BAT and Liver measured at Day 0 (Control no cold exposure), Days 1, 5 and 10 post cold treatment at 4 ^0^ C (*n* = 2 for all groups). **A**: ANGPTL8 protein expression in SAT at different time points of cold treatment. **B**: ANGPTL8 protein expression in BAT at different time points of cold treatment. **C**: ANGPTL8 protein expression in Liver at different time points of cold treatment

**Fig. 6:**
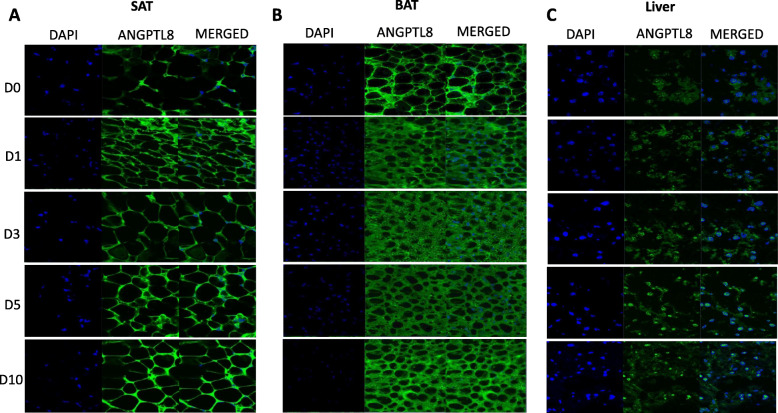
Representative Immunohistochemistry staining of ANGPTL8 protein level in SAT, BAT and Liver measured at Day 0 (Control no cold exposure), Days 1, 3, 5 and 10 post cold treatment at 4 ^0^ C (*n* = 3 for all groups). **A**: ANGPTL8 protein expression in SAT at different time points of cold treatment. **B**: ANGPTL8 protein expression in BAT at different time points of cold treatment. **C**: ANGPTL8 protein expression in Liver at different time points of cold treatment

To confirm the ANGPTL8 gene expression data, WB and IHC were performed to measure the protein levels of ANGPTL8. The results obtained by WB analysis indicated an increase in ANGPTL8 protein levels in SAT on Day 10 (Fig. 5 A). No change was observed in ANGPTL8 protein levels in BAT (Fig. 5B**).** However, in the liver, ANGPTL8 protein levels decreased on Days 1 and 5, followed by a subsequent normalization of the protein levels on Day 10 of cold exposure compared with Day 0 (Fig. 5 C). Similarly, IHC was used to evaluate ANGPTL8 protein levels in SAT, BAT, and liver tissues obtained from mice exposed to cold treatment compared with corresponding tissue sections obtained from control mice (Fig. 6). The results indicated an increase in ANGPTL8 protein levels in SAT with chronic cold treatment (Fig. 6 A). In contrast, no change was observed in ANGPTL8 protein levels in BAT (Fig. 6B), whereas a decrease in ANGPTL8 protein levels was observed in the liver tissue (Fig. 6 C).

## Discussion

In this study, the expressions of ANGPTL3, 4, and 8 in the liver, SAT, and BAT were investigated under acute and chronic cold conditions. In the liver, ANGPTL3 and 8 expressions decreased after acute cold treatment, and this decrease was maintained throughout the course of the 10-day treatment. In contrast, ANGPTL4 expression in the liver was not affected by cold treatment. In SAT, ANGPTL8 showed increased expression after cold treatment, whereas ANGPTL4 expression was reduced. Although the highest increase in ANGPTL8 gene expression was observed on Day 1 of cold treatment, at the protein level, the increase was highest on Day 10. This could indicate that the increase in ANGPTL8 protein levels under chronic cold conditions may be the result of a posttranslational mechanism, warranting further investigation.

As expected, *UCP1* gene expression in SAT was markedly increased after acute cold treatment and this increase was maintained even under chronic cold conditions. Interestingly, *LPL* and *ATGL* gene expression were increased under acute cold treatment, followed by a steady decrease over time. Similar gene expression results for *UCP1*, *LPL* and *ATGL* were previously reported in WAT following 24 h of cold exposure at 4˚C [18]. This agrees with the data from the current study. However, long term effects of cold exposure on the expression of these genes have not been extensively studied.

In BAT, ANGPTL4 expression was significantly reduced, whereas ANGPTL8 expression was unchanged throughout the treatment. Previous studies reported a reduction in ANGPTL4 expression during acute and chronic cold exposure. This is important as it results in increased LPL activity in BAT [3, 16, 17]. The data presented in this study corroborate these findings and further support the role of ANGPTL4 in BAT during chronic cold treatment. Furthermore, *UCP1* gene expression was significantly increased following acute cold conditions. This increase was followed by a reduction in expression to normal levels over the course of the treatment. *LPL* gene expression did not change with cold treatment. However, *ATGL* gene expression was significantly reduced after acute cold treatment, and there was a slight increase with chronic cold treatment. Because ANGPTL3 is exclusively expressed in the liver, its expression was not evaluated in SAT and BAT.

### Comparisons with other studies and new findings

The data obtained from the current study reveal a parallel increase in the gene expressions of *ANGPTL8* and *UCP1* under acute and chronic cold conditions. Interestingly, following acute cold treatment, a parallel increase in gene expression was also observed between *ANGPTL8* and the genes associated with lipolysis (*ATGL* and *LPL*), and an inverse relationship with the level of these genes under chronic cold conditions.

Among the many roles that WAT plays in physiology, its role as an energy store during cold treatment is critical for directing lipids toward BAT for thermogenesis. The response of adipose tissue to cold demonstrates the highly dynamic and elastic nature of this tissue [9, 14, 19]. For instance, when exposed to extended cold treatment, WAT differentiates into beige/brite adipocytes. Similar to BAT, beige cells exhibit high levels of UCP1 and many mitochondria exhibiting high thermogenic capacity [20]. Through a series of experiments involving the knockdown and overexpression of ANGPTL8, it was demonstrated that ANGPTL8 can significantly promote lipid deposition and adipocyte differentiation [21]. Furthermore, ANGPTL8 forms a complex with either ANGPTL3 or ANGPTL4. As a complex, ANGPTL8/ANGPTL3 or ANGPTL8/ANGPTL4 can differentially regulate LPL activity, which in turn regulates lipolysis [7]. These studies indicate the importance of ANGPTL8 in both adipocyte differentiation and lipolysis. The data from the current study showed that the expression of ANGPTL8 in SAT was induced by chronic cold treatment, thereby highlighting the significance of ANGPTL8 in mechanisms that are critical during cold treatment, such as lipolysis. It is possible that the differential expressions of ANGPTL8 and 4 in SAT observed in the current study play a role in this process. Furthermore, the data shed light on an inverse relationship between the level of ANGPTL8 and the genes involved in lipolysis under chronic cold conditions. This further supports the suggestion that one of the functions of ANGPTL8 under cold conditions is the inhibition of lipolysis. This is a beneficial mechanism, as it has been proposed that an increase in lipolysis in WAT leads to increased levels of FAs in circulation. These FAs are then taken up by the liver and other ectopic tissues and could result in complications such as nonalcoholic fatty liver disease and insulin resistance [22, 23]. Furthermore, the increased expression of ANGPTL8 concomitant with the expression of UCP1 indicates a potential secondary role of ANGPTL8 in SAT. Considering that UCP1 is associated with increased browning of WAT, the parallel increase in ANGPTL8 suggests that it may be associated with the browning of SAT. This remains to be validated in a tissue-specific knockout model along with further experimentation.

The liver plays a major role in energy homeostasis by regulating glucose and lipid metabolic pathways. It is the major source of ANGPTL3 and ANGPTL8, which are well known regulators of LPL activity [24–26]. During extended cold exposure, ANGPTL3 and ANGPTL8 are inhibited in the liver to maintain high LPL activity and direct FAs toward BAT for heat generation. In the present study, a decrease was observed in liver ANGPTL3 and ANGPTL8 expression levels, which agrees with the expected outcome. However, ANGPTL4 expression in the liver was maintained throughout the cold treatment. This may be explained by the fact that ANGPTL4 is induced mainly by fasting and the animals were in a fed state.

As expected, the data obtained from gene expression analysis in BAT tissue, indicates the importance of this tissue in rodents in response to cold conditions. A previous study showed that knocking out the UCP1 in mice leads to enhanced sensitivity of the animals to cold temperatures[27]. Therefore, the increase in *UCP1* expression level seen under acute cold conditions, further supports the importance of this gene. Furthermore, the reduction in the expression level of this gene over the course of time in cold conditions suggests the possibility of a switch in the mechanism associated with metabolic changes in BAT. A recent study showed that cold conditions induce UCP1 dependent/independent thermogenesis in BAT [28]. The reduction in the expression of ATGL under acute cold conditions further supports that under acute cold conditions the process of lipolysis in BAT is not dependent on ATGL[29]. It is possible that in BAT, the inhibition of ANGPTL4 leading to an increased LPL activity is the primary metabolic mechanism in play under cold conditions.

In summary, the data from this study agrees with previously published reports. The novelty lies in highlighting the importance of ANGPTL8, specifically in SAT, under acute and chronic cold conditions.

## Strengths and limitations of the study

In this study, the gene expression levels of markers associated with lipid metabolism and energy homeostasis were investigated for the first time under both acute and chronic cold conditions. The strength of the obtained data lies in the number of animals in each study group, which contributed toward obtaining data with statistical significance. Moreover, the variation between the results from each group was considerably low, adding to the study’s strength. In addition, the data were obtained from different tissues and confirmed using multiple approaches.

One of the limitations of this study was the design of the experiment to have food available throughout cold treatment. Under these conditions, the nonshivering thermogenesis energy source in BAT can be either FAs or carbohydrates from food. Under fasting conditions, FAs become the main energy source through ATGL-mediated lipolysis. However, because of the extended cold treatment, animals will not survive without food. Owing to the unavailability of metabolic chambers, whole-body energy metabolism parameters, including core body temperature, oxygen consumption (VO_2_), carbon dioxide production (VCO_2_), and physical activity, during the cold exposure period could not be assessed. This would provide a better explanation of the obtained data as there was a significant increase in food consumption (Fig. 1B), a significant decrease in the total SAT weight (Fig. 1 C), and a significant increase in the average BAT weight (Fig. 1D) in mice following cold exposure compared with control mice. Another limitation of this study is that WB analysis was performed only on ANGPTL8 in all tissues. This was because the novelty of this study was to highlight the importance of ANGPTL8 and confirm the data obtained from gene expression analysis for this protein. Moreover, the data from the study is based on checking mRNA levels of various genes across different tissues. Inferences on the importance of ANGPTL8 are made through its association with genes known to be involved in browning and lipolysis under cold conditions.

## Conclusions

The data from this study draw attention to the potential role of ANGPTL8 in SAT under acute and chronic cold conditions. This may be through the regulation of lipolysis and SAT browning. Consistent with previous studies, the data also show a reduction in ANGPTL4 in BAT, which is known to improve thermogenesis in response to cold exposure. This study further highlights the complexity of the interaction between the ANGPTL proteins and their importance in regulating lipid metabolism during normal and stressful conditions. Overall, the findings of this study demonstrate the potential of targeting ANGPTL proteins for regulating the mechanisms underlying lipolysis and browning in SAT. This could consequently be used to combat metabolic disorders.

## Supplementary information

Additional file 1 Supplementary Figure 1: The uncut images of the Western blotting membranes of ANGPTL8 protein level in SAT, BAT and Liver.

## Data Availability

Data is available upon request.

## Abbreviations

ANGPTL: Angiopoietin-like protein
WAT: White adipose tissue
BAT: Brown adipose tissue
SAT: Subcutaneous adipose tissue
LPL: Lipoprotein lipase
TG: Triglyceride
UCP1: Uncoupling protein 1
VLDL: Very low-density lipoprotein
CV: Coefficients of variation
