# Supplementary figures and images for: Increased expression level of ANGPTL8 in white adipose tissue under acute and chronic cold treatment
Source: Lipids Health Dis. 2021 Sep 26;20:117. doi: 10.1186/s12944-021-01547-0 (PMC8466641; doi:10.1186/s12944-021-01547-0)

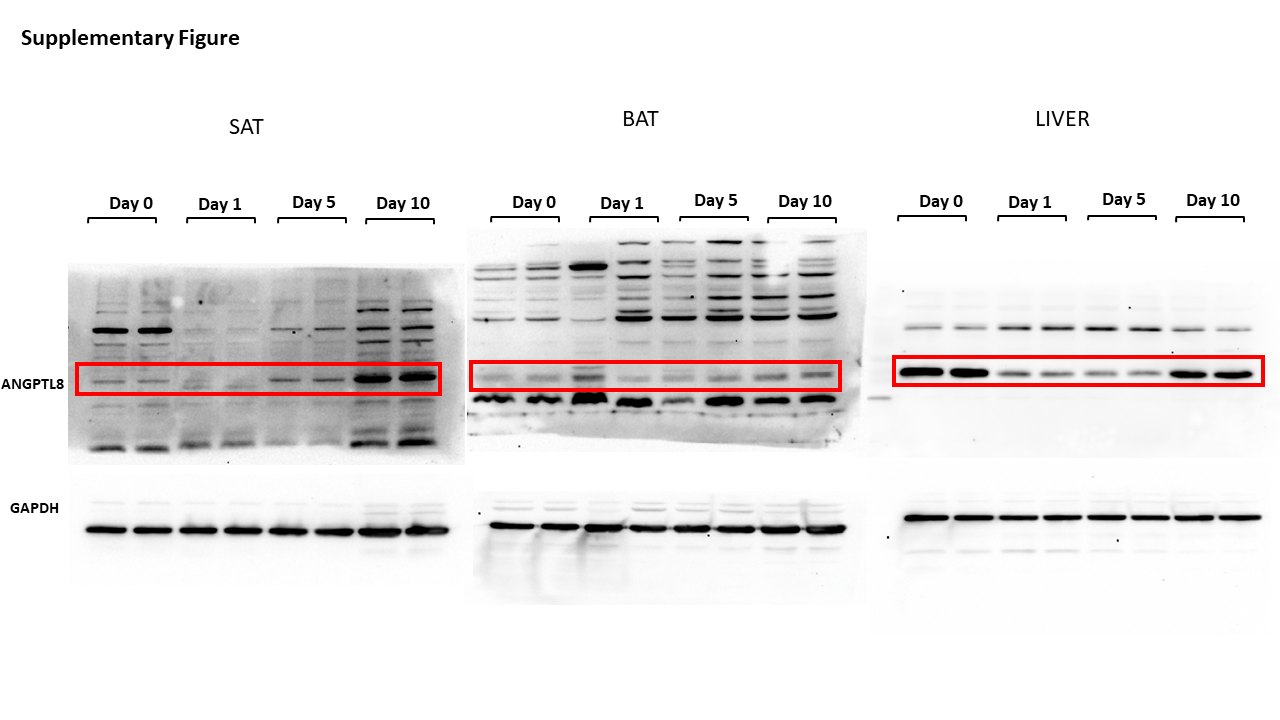

Supplement: Supplementary file 1 — Supplementary Figure 1: The uncut images of the Western blotting membranes of ANGPTL8 protein level in SAT, BAT and Liver. [file 12944_2021_1547_MOESM1_ESM.tif]
